# Fatal Outcome of Ilheus Virus in the Cerebrospinal Fluid of a Patient Diagnosed with Encephalitis

**DOI:** 10.3390/v12090957

**Published:** 2020-08-29

**Authors:** Bruno H. G. A. Milhim, Cássia F. Estofolete, Leonardo C. da Rocha, Elisabete Liso, Vânia M. S. Brienze, Nikos Vasilakis, Ana C. B. Terzian, Maurício L. Nogueira

**Affiliations:** 1 Laboratório de Pesquisas em Virologia (LPV), Faculdade de Medicina de São José do Rio Preto (FAMERP), Avenida Brigadeiro Faria Lima, 5544 - Vila Sao Jose, 15090-000 São José do Rio Preto, Brazil; brunohgam@hotmail.com (B.H.G.A.M.); cassiafestofolete@gmail.com (C.F.E.); leocecilior@hotmail.com (L.C.d.R.); anacarolinaterzian@gmail.com (A.C.B.T.); 2 Hospital de Base, Avenida Brigadeiro Faria Lima, 5544 - Vila Sao Jose, SP 15090-000 São José do Rio Preto, Brazil; eliliso@terra.com.br (E.L.); vania.brienze@hospitaldebase.com.br (V.M.S.B.); 3 Department of Pathology, University of Texas Medical Branch, 301 University Blvd., Galveston, TX 77555-0609, USA; nivasila@utmb.edu; 4 Center for Biodefense and Emerging Infectious Diseases, University of Texas Medical Branch, 301 University Blvd., Galveston, TX 77555-0609, USA; 5 Center for Tropical Diseases, University of Texas Medical Branch, 301 University Blvd., Galveston, TX 77555-0609, USA; 6 Institute for Human Infection and Immunity, University of Texas Medical Branch, 301 University Blvd., Galveston, TX 77555-0610, USA

**Keywords:** Ilheus virus, cerebrospinal fluid, atypical manifestations, cerebrovascular event

## Abstract

Ilheus virus is an arbovirus with the potential for central nervous system involvement. Accurate diagnosis is a challenge due to similar clinical symptoms and serologic cross-reactivity with other flaviviruses. Here, we describe the first documented case of a fatal outcome following the identification of Ilheus virus in the cerebrospinal fluid (CSF) of a patient with cerebral encephalitis in Brazil.

## 1. Introduction

Ilheus virus (ILHV) is an arbovirus that was first described in 1944 and isolated from *Aedes* and *Psorophora spp*. mosquitoes during an epidemiological investigation of yellow fever in the city of Ilheus, Bahia State, Brazil [1,2]. ILHV belongs to the genus *Flavivirus*, family *Flaviviridae* in the Ntaya antigenic complex. ILHV is maintained in an enzootic transmission cycle between birds and arboreal mosquitoes belonging to eight genera (*Aedes, Culex, Coquillettidia, Haemagogus, Sabethes, Trichoprosopon*, *Psorophora*, and *Ochlerotatus*, with the last two being considered the primary vectors of transmission). Since its initial isolation, ILHV has been isolated or detected primarily in arboreal mosquitoes [1,3,4,5,6,7,8,9], birds [10,11], and humans [8,12,13,14,15,16,17,18,19] throughout Central America (Honduras, Guatemala, and Panama), the Caribbean (Trinidad and Tobago), and South America (Argentina, Bolivia, Brazil, Colombia, Ecuador, French Guyana, Peru, and Venezuela), suggesting a broad geographic range of transmission. Various serological surveys showed the presence of ILHV-neutralizing antibodies in rodents [20], coatis [10], birds [6,10,20,21], horses [22,23], sentinel [8] and wild monkeys [10,20,24,25,26,27], and humans [1,17,18,20,28,29].

Since 2006, we have established arbovirus surveillance for encephalitis-suggestive cases in the city of São José do Rio Preto (SJdRP), State of São Paulo, Brazil, located in a hyper-endemic area for dengue virus (DENV) [30,31,32,33,34,35,36,37,38,39], Saint Louis encephalitis [40,41], Zika virus (ZIKV) [34], and documented co-infection among various flaviviruses [30,41,42]. Herein, we report a case of cerebral encephalitis in a patient infected with ILHV that was observed by our surveillance team in São José do Rio Preto (SJdRP).

## 2. Materials and Methods

### 2.1. Ethics Statement

This study was submitted and approved by the Ethical Review Board (process number 15461513.5.0000.5415, 24 May 2019) of the School of Medicine of São José do Rio Preto (FAMERP), São Paulo, Brazil. Confidentiality was ensured by de-identifying all questionnaires and samples before the data entry and analysis.

### 2.2. Medical History and Sample Collection

Through an arbovirus surveillance program established in SJdRP, all dengue-suspected cases with warning signs (DwWS) or severe disease (SD) were monitored by our team from admission to discharge. The classification of warning signs includes abdominal pain or tenderness, persistent vomiting, clinical fluid accumulation, mucosal bleed, lethargy, restlessness, liver enlargement >2 cm, and an increase in the hematocrit concurrent with a rapid decrease in platelet count. Severe dengue disease is classified by the presence of severe plasma leakage (shock and fluid accumulation with respiratory distress), severe bleeding, and/or severe organ involvement (aspartate aminotransferase and/or alanine aminotransferase ≥1000 UI/L, impaired consciousness, and heart and other organ failure). This case was part of a hospital-based retrospective and descriptive study conducted with biobanked cerebrospinal fluids from patients with central nervous system (CNS) impairment observed between January 2016 and December 2017. During the same period, there were 16,898 laboratory-confirmed dengue cases in the city (DENV RT-PCR positive, NS1 antigen detection reagent, and/or anti-dengue immunoglobulin type M (IgM) reagent), of which, 287 were further screened and had clinical samples (notably, CSF) submitted retrospectively for further diagnostic tests for arbovirus detection at the Laboratório de Pesquisas em Virologia (LPV), located within FAMERP. Demographic, epidemiological (gender and age), and clinical data (symptoms and radiologic observations) were obtained from electronic records and reported to the medical team.

### 2.3. Diagnostic Analyses

Due to the limited quantity of the CSF samples available, we opted not to attempt virus isolation. Samples were submitted for virus RNA (vRNA) extraction using the Kit QIAmp^®^ Viral RNA (QIAGEN^®^, Germantown, MD, USA) following the manufacturer’s recommendations. The Trioplex quantitative polymerase chain reaction (qPCR) assay was performed using a kit provided by the Centers for Disease Control and Prevention using primers and probes specifically designed for the detection of ZIKV, chikungunya virus (CHIKV), and all DENV serotypes [43]. A total of 10 µL of vRNA, 0.5 µM of each probe and primer mixed, 12.5 µL of the 2X PCR Master Mix, and 0.5 µL of Superscript III RT/Platinum Taq enzyme mix (SuperScript^®^ III Platinum^®^ One-Step qRT-PCR System, Invitrogen, Carlsbad, CA, USA) were applied to a 96-well plate using the QuantStudio™ Dx instrument (Thermo Fisher Scientific, Waltham, MA, USA) with the following conditions: 50 °C for 30 s, followed by 45 cycles of 95 °C for 15 s, and 60 °C for 1 min. The results were interpreted as being positive when the cycle threshold (Ct) values were less than 38.

The duplex-nested PCR assay targeting a conserved domain of the nonstructural protein 1 (nsp1) and 5 (NS5) genes that was designed for the detection and identification of Brazilian alphaviruses and flaviviruses, respectively [44], was also utilized. An amplicon that was 401 nucleotides long was obtained, purified, and sequenced via the Sanger method [45] using a Big Dye Terminator Kit v3.1 (Applied Biosystems, Foster City, CA, USA) according to the manufacturer’s instructions, and deposited in GenBank (MK266240). The obtained sequence was analyzed using BLAST (www.ncbi.nlm.nih.gov/blast/Blast.cgi) software (v1.4.0) and the alignment to known GenBank sequences. To eliminate any chance of contamination, a strict processing protocol was established based on the unidirectional flow of processes, including separate rooms for pre-PCR sample preparation, RNA extraction, PCR assembly, and running steps, with intensive use of individual protection equipment. Additionally, a rigorous cleaning and decontamination protocol of all surfaces and equipment (20 min contact time with 70% ethyl alcohol and exposure to ultraviolet light) was performed throughout the process. For this study, the ILHV genetic material was not used at any time during this process as a positive control, further reducing the possibility of contamination.

### 2.4. Phylogenetic Analysis

The ILHV evolutionary history was inferred using the maximum likelihood method based on the general time-reversible model with a bootstrap of 1000 replicates [46] and using a dataset of 13 representative ILHV strains with a spatiotemporal span of 73 years (1944–2017) comprising 401-nucleotide-long sequence mapping on the NS5 gene. The tree with the highest log-likelihood (−1087.95) was found. Initial tree(s) for the heuristic search were obtained automatically by applying the Neighbor-Join and BioNJ algorithms to a matrix of pairwise distances that were estimated using the maximum composite likelihood (MCL) approach and then selecting the topology with a superior log-likelihood value. A discrete gamma distribution was used to model the evolutionary rate differences between sites (five categories (+G, parameter = 0.8348)). The rate variation model allowed for some sites to be evolutionarily invariable ([+I], 52.27% sites). The tree was drawn to scale, with branch lengths measured in the number of substitutions per site. A homologous nucleotide sequence from Rocio virus (ROCV) was used as an outgroup to root the ILHV tree. All positions containing gaps and missing data were eliminated, leaving a total of 401 positions in the final dataset. Evolutionary analyses were conducted in MEGA7 [47].

## 3. Results

**Figure 1:**
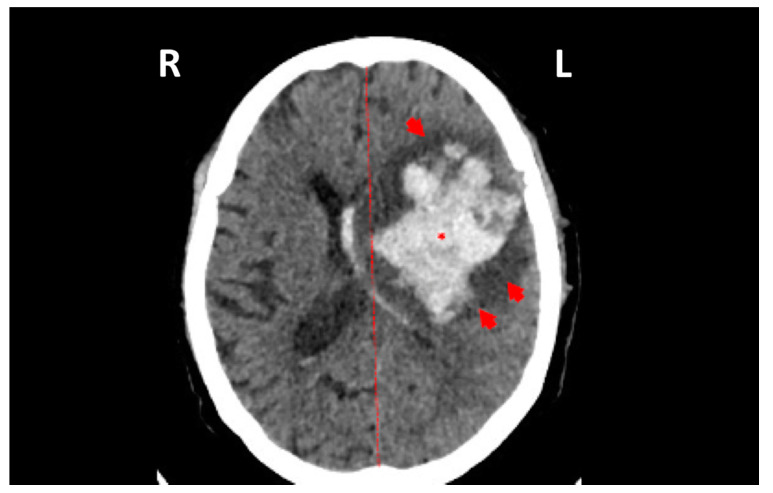
Computed tomography imaging of the patient’s brain showing an intraparenchymal hemorrhage (asterisk) surrounded by a brain edema (red arrows), as well as deviation from the cerebral middle line (red line).

Between January 2016 and December 2017, 287 CSF samples were evaluated for possible arbovirus infection; in one of those, the genetic sequence of ILHV was detected by the highly specific PCR assay. The sample corresponds to a 68-year-old man who was admitted on 28 September 2017 with right hemiplegia, aphasia, dysarthria, and deviation of the left lip rhyme. He suffered from blood hypertension and diabetes. During hospitalization, brain computed tomography showed an intraparenchymal hemorrhage in the parietal lobe, surrounding brain edema, and a deviation from the cerebral middle line (Figure 1). He exhibited no symptoms of infection until admission and had no travel history. Cerebrospinal fluid was collected (leukocytes 840 cells/mm^3^ (83% lymphomonocytes), glucose 57 mg/dL, protein 338 mg/dL, and bacteria culture negative) and he underwent bleeding drainage. After 6 days of drainage, testing of the CSF showed leukocytes at 190 cells/mm^3^ (80% lymphomonocytes), glucose 102 mg/dL, protein 802 mg/dL, and the bacteria culture negative. Subsequently, the patient developed a urinary infection and pneumonia from multi-drug resistant bacteria (*E. coli* and *Acinetobacter baumannii,* respectively) and died 24 days following admission.

**Figure 2:**
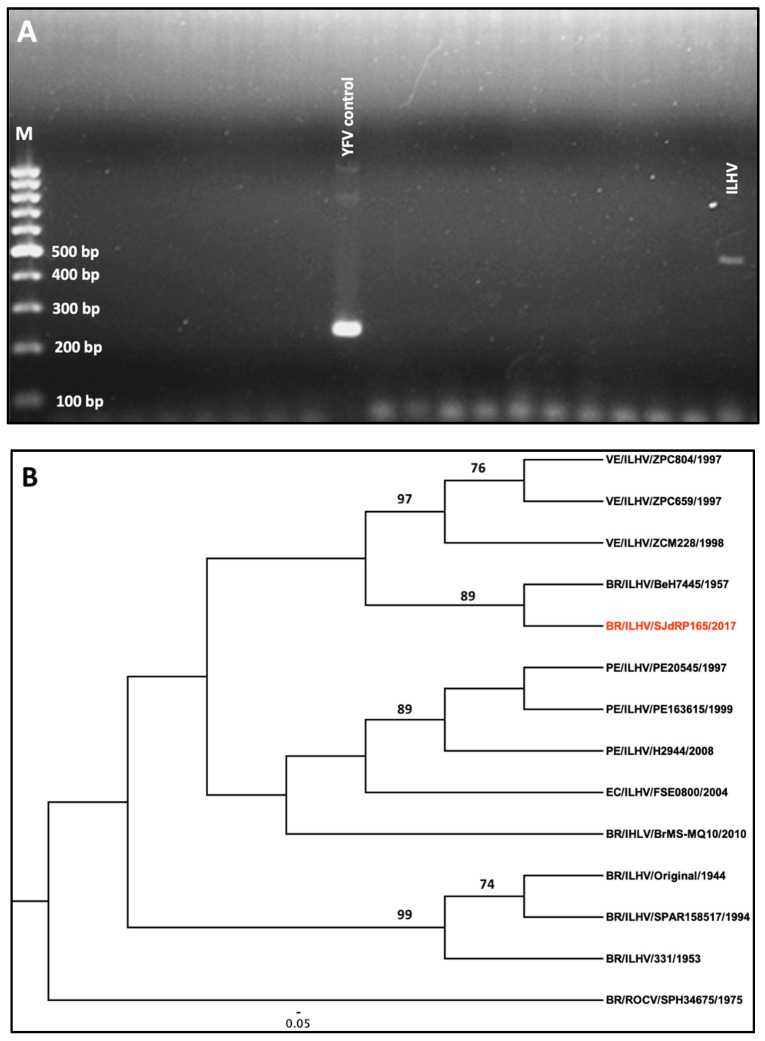
(**A**) Molecular detection of the Ilheus virus (ILHV) partial sequence of the NS5 gene on a 1.5% agarose gel using yellow fever virus (YFV) as a positive control. (**B**) Phylogeny of Ilheus virus inferred using the maximum likelihood method. The maximum likelihood tree was obtained from a sequence dataset of 11 isolates using a General Time Reversible (GTR) substitution model. Branches are labeled with bootstrap values that represent the percentage of 1000 replicates in which the members of a given clade were predicted to relate in the same topography. The scale shows a genetic distance of 0.05, or a 5% nucleotide sequence divergence. A homologous sequence from Rocio virus (ROCV) was used as an outgroup to root the ILHV tree. The sequence of the strain for this study is indicated in red. Abbreviations: VE—Venezuela; BR—Brazil; PE—Peru; EC—Ecuador.

A CSF sample was also submitted for RNA extraction and tested for dengue, Zika, and chikungunya viruses using the Trioplex quantitative polymerase chain reaction (qPCR) [43] assay, as well as using an assay designed to detect several other arboviruses endemic to Brazil with primer sets that are specific for conserved domains of the gene encoding for the RNA-dependent RNA-polymerase (RdRp) [44]. The resulting amplicons that were 401 nucleotides long were sequenced and ILHV was the only arbovirus identified (Figure 2a). Phylogenetic analysis demonstrated clustering with isolates sampled in Venezuela in 1997, suggesting the widespread distribution of the virus throughout Latin America (Figure 2b).

## 4. Discussion

Current knowledge suggests that ILHV is not associated with any known epidemics, and human infection has been sporadically reported in Trinidad [15], Panama [16], Colombia [14], French Guyana [13], Brazil [8,48], Ecuador [12], and Bolivia [19]. The clinical spectrum of human infections ranges from asymptomatic to severe disease that is characterized by central nervous system involvement that is suggestive of encephalitis. Viremia lasts three to five days and most patients exhibit a mild febrile illness, accompanied by headache, myalgia, photophobia, and arthralgia, as well as non-specific symptoms that may suggest dengue fever, yellow fever, or influenza [8,15,49]. CNS involvement has been observed infrequently in the handful of documented human cases and may indicate progression to severe Ilheus disease, whose clinical presentation is suggestive of viral encephalitis ([15,48,50] and Table 1). Furthermore, the few clinically diagnosed cases documented to date (Table 1) contrast with the observed prevalence of ILHV antibodies in humans [1,17,18,20,28,29], suggesting that most infections are inapparent or misdiagnosed. Mild non-specific symptoms, short viremia, high levels of antibodies showing cross-reactivity with flaviviruses, and a lack of routine laboratory assays are some of the barriers that may complicate an accurate ILHV diagnosis [19].

Given the shortage of data in the literature on neurological events in ILHV infections, it is reasonable to propose that physicians could leverage the acquired knowledge from other well-characterized flavivirus infections, such as DENV, Japanese encephalitis virus (JEV), and West Nile virus (WNV). Our observations may not conclusively demonstrate that ILHV infection led to the brain hemorrhage and death in a patient with an acute neurological syndrome and underlying conditions (diabetes and hypertension). Considering the increasing incidence of arboviruses with unusual manifestations ([51,52,53] and reviewed in [54,55,56]), and their potential tropism for the invasion of the CNS, leading to long-term neurofunctional sequelae, this report highlights the need for vigilance among physicians, healthcare providers, and researchers alike for arbovirus infections in patients presenting with meningoencephalitis and cerebrovascular events. Furthermore, this study demonstrates the importance of comprehensive arbovirus surveillance beyond urban arboviruses (e.g., DENV, CHIKV, and ZIKV), suggesting that urban and peri-urban populations may be at risk for ILHV infection and other emerging zoonotic arboviruses. Given the high level of antibody cross-reactivity with flaviviruses and the lack of routine laboratory serological assays, which complicates an accurate diagnosis of arboviruses, this poses an urgent call for employing new and affordable technologies [57,58] for the development of more accurate diagnostic assays

## Figures and Tables

**Table 1:** Clinical symptoms that were observed in documented ILHV human cases.

Country, Year	Number of Cases	Clinical Symptoms	Diagnostic Tests Performed	Reference
USA, 1950	19	- Experimental infection in cancer patients for the clinical characterization of the ILHV infection. Viremia was observed in 9 out of the 19 patients. Fever, malaise, muscle pain, lethargy, somnolence, and mild encephalitis was observed in three out of the nine symptomatic patients.	Blood and serology testing (HI, CF, mouse neutralization test)	[50]
Brazil, 1957–1959	2	- Male, presented with a fever, headache, dizziness, muscle pain, and weakness. - Female, presented with headache, dizziness, muscle and joint pain and weakness, photophobia, and nausea.	Blood and serology testing (HI, CF, mouse neutralization test)	[8]
Trinidad, 1955–1957	3	- 37-year-old female, presented with a fever, headache, photophobia, joint and muscle pain, nausea, cough, constipation, and transient neurological involvement (diplopia). - 18-year-old male, asymptomatic infection. - A 22-year-old male presented with acute febrile syndrome (fever, malaise, and chills).	Blood and serology testing (HI, CF, mouse neutralization test)	[15]
Panama, 1964	1	- Male, presented with fever and headache.	Blood and serology testing (HI)	[16]
Colombia, 1966	1	- 28-year-old male, asymptomatic whose serum was obtained during an epidemiologic study of a series of suspected cases of infectious hepatitis in a penal colony.	Blood and serology testing (HI, CF, mouse neutralization test)	[14]
French Guiana, 1973	1	- Male, presented with mild “dengue-like” syndrome.	Blood and serology testing (HI, CF)	[13]
Brazil, 1995	5 *	- 24-year-old male, presented with syndrome fever, vomiting, headache, diarrhea, dry cough, fatigue, and dyspnea. The patient also presented with swollen ganglia in the axillary, cervical, inguinal, and epitrochlear regions. - 22-year-old male, presented with a fever, myalgia, arthralgia, cough, polyuria, diarrhea, and abdominal pain. - 22-year-old male, presented with a fever and a dry cough. - 20-year-old male, presented with a fever, diarrhea, somnolence, dizziness, tremors, dysarthria, ataxia, headache, mental confusion, facial paralysis, strabismus, and viral encephalitis. - 72-year-old male, presented with a fever, sweating, cramps, hepatomegaly, headache, adynamia, anorexia, and papulolenticular exanthema.	Blood and serology testing (HI, CF, mouse neutralization test)	[48]
Ecuador, 2004	1	- 20-year-old male, presented with a fever, rash, epistaxis, headache, myalgia, retroocular pain, nausea, vomiting, jaundice, sore throat, and abdominal pain.	Blood work	[12]
Bolivia, 2005	1	- 15-year-old male, presented with a fever, malaise, asthenia, conjunctival injection, rash, arthralgia, myalgia, abdominal pain, headache, and earache. No signs of cardiac, neurological, or renal damage were detected.	Blood, molecular (RT-PCR) and serology (IgM ELISA) testing	[19]
Brazil, 2017	1	- 68-year-old male, presented with right hemiplegia, aphasia, dysarthria, deviation of the left lip rhyme, and viral encephalitis.	Molecular testing of CSF (qPCR)	Present paper

Abbreviations: HI—hemagglutination inhibition, CF—complement fixation, RT-PCR—reverse transcription polymerase chain reaction, IgM ELISA—immunoglobulin M enzyme-linked immunosorbent assay. (*) Although the virus was isolated from the serum of each patient, it was not sequenced for confirmation.
